# Multimorbidity and Frailty Are the Key Characteristics of Patients Hospitalized with COVID-19 Breakthrough Infection during Delta Variant Predominance in Italy: A Retrospective Study

**DOI:** 10.3390/jcm11185442

**Published:** 2022-09-16

**Authors:** Andrea Ticinesi, Alberto Parise, Nicoletta Cerundolo, Antonio Nouvenne, Beatrice Prati, Giulia Chiussi, Angela Guerra, Tiziana Meschi

**Affiliations:** 1 Department of Medicine and Surgery, University of Parma, Via Antonio Gramsci 14, 43126 Parma, Italy; 2 Geriatric-Rehabilitation Department, Azienda Ospedaliero-Universitaria di Parma, Via Antonio Gramsci 14, 43126 Parma, Italy

**Keywords:** SARS-CoV-2, vaccine failure, viral pneumonia, geriatric patient, comorbidity

## Abstract

The aims of this study were to describe the characteristics of patients hospitalized with delta SARS-CoV-2 breakthrough infection, and to identify factors associated with pneumonia on chest Computed Tomography (CT) and mortality. The clinical records of 229 patients (105 F), with a median age of 81 (interquartile range, IQR, 73–88) years old, hospitalized between June and December 2021 after completion of the primary vaccination cycle, were retrospectively analyzed, retrieving data on comorbidities, Clinical Frailty Scale (CFS), clinical presentation and outcomes. Multimorbidity (91.7% with ≥2 chronic illnesses) and frailty (61.6% with CFS ≥ 5) were highly prevalent. CFS (OR 0.678, 95% CI 0.573–0.803, *p* < 0.001) and hypertension were independently associated with interstitial pneumonia. Mortality was 25.1% and unrelated with age. PaO_2_/FiO_2_ on blood gas analysis performed upon admission (OR 0.986, 95% CI 0.977–0.996, *p* = 0.005), and CFS (OR 1.723, 95% CI 1.152–2.576, *p* = 0.008) were independently associated with mortality only in subjects < 85 years old. Conversely, serum PCT levels were associated with mortality in subjects ≥ 85 years old (OR 3.088, 95% CI 1.389–6.8628, *p* = 0.006). In conclusion, hospitalization for COVID-19 breakthrough infection mainly involved geriatric patients, with those aged ≥ 85 more characterized by decompensation of baseline comorbidities rather than typical COVID-19 respiratory symptoms.

## 1. Introduction

Mass vaccination campaigns against Severe Acute Respiratory Syndrome-Coronavirus 2 (SARS-CoV-2) have substantially modified the clinical and epidemiologic characteristics of Coronavirus Disease-19 (COVID-19) [1]. Vaccines have shown a good, though incomplete, capacity of hindering SARS-CoV-2 transmission by reducing viral loads and duration of viral shedding [2,3] and have substantially mitigated the burden of COVID-19 symptoms, namely fever and dyspnea, in subjects with breakthrough infection [4].

Several studies have confirmed that the clinical course and outcomes of COVID-19 are substantially different in vaccinated subjects, with lower risk of hospital admission, progression to severe disease, need of oxygen or ventilatory support, and death [5,6,7,8,9,10,11,12,13]. In the earliest phases of the vaccination campaign, these results were also reported for older patients [6], whose high burden of chronic illnesses and frailty was associated with increased risk of severe COVID-19 course in the pre-vaccination era [14,15].

However, a population study based in England has recently shown that SARS-CoV-2 vaccine breakthrough infections may still retain substantial clinical severity and risk of adverse outcomes in selected high-risk groups, including older, immunocompromised or dialyzed subjects [16]. A nation-wide study from Scotland pointed out that severe course of COVID-19 breakthrough infection, requiring hospitalization, was significantly associated with age ≥ 80 years old, ≥5 chronic diseases and previous hospital admission for other reasons [17]. Several conditions frequently present in older age, including diabetes [18], cancer [19] and dementia [20], have also been associated with increased risk of breakthrough SARS-CoV-2 infection and clinical severity.

Interestingly, in a recent systematic review and meta-analysis of studies comparing the clinical manifestations of COVID-19 between vaccinated and unvaccinated subjects, breakthrough infection was not associated with reduced risk of hospitalization, invasive ventilation or mortality [21]. This apparently puzzling result could however be influenced by changes of the characteristics of patients requiring hospitalization for COVID-19, with more subjects with pre-existing frailty and multimorbidity and more admissions for reasons unrelated to SARS-CoV-2 infection [22].

In the second half of 2021, concomitantly with the surge of the SARS-CoV-2 delta variant, breakthrough infections have accounted for a substantial and increasing portion of COVID-19 hospitalizations in developed countries and particularly in Italy, due to the high rates of vaccination reached among the general population [23]. In spite of this, few reports have systematically described the clinical characteristics of these patients in terms of comorbidities, frailty, clinical presentation and care needs during hospital stay.

The objective of this retrospective single-center study was thus to describe the clinical features, outcomes and care needs of patients admitted with COVID-19 breakthrough infection in a large regional hospital in Northern Italy in the period of maximum circulation of the delta variant (June–December 2021), and to identify factors associated with the presence of interstitial pneumonia and adverse outcome.

## 2. Materials and Methods

### 2.1. Study Design, Setting, and Population

The study was conducted at the Internal Medicine unit of the Geriatric-Rehabilitation Department of Parma University-Hospital, in Northern Italy. Since the earliest phases of the first pandemic wave, this unit was converted into a COVID-19 unit, serving a catchment area of around 450,000 inhabitants (province of Parma, Emilia-Romagna region) [24]. In 2021, the main criterion for admission to this unit was a positive reverse transcriptase-polymerase chain reaction (RT-PCR) test for SARS-CoV-2 on nasopharyngeal swabs. Thus, admitted patients had COVID-19, but did not necessarily have respiratory involvement and the main reason of hospitalization could be, in some cases, unrelated to COVID-19.

Included in this retrospective study were all patients ≥ 18 years old who were admitted between 1 June and 31 December 2021 (period of maximum diffusion of the delta variant in Italy) with positive RT-PCR test and had completed the primary anti-SARS-CoV-2 vaccination cycle more than 14 days before admission (i.e., two doses of mRNA BNT162b2, mRNA-1273 or ChAdOx1-S vaccines, one dose of Ad26.COV2.S vaccine). In subjects with previous COVID-19 infection, the primary vaccination cycle was considered completed 14 days after receiving the first dose of any vaccine. Subjects who had already received a third “booster” dose of vaccine, recommended in Italy to over 65 and frail subjects from September 2021 and to all the general population from November 2021, were included as well.

All subjects who were vaccinated with other vaccines not approved for human use in the European Union, refused to complete the primary cycle after receiving a first dose, were exempted from vaccination for medical reasons, or completed the primary vaccination cycle in the fourteen days preceding admission were excluded from the study. Informed consent denial for data treatment was also another exclusion criterion.

### 2.2. Data Collection and Study Endpoints

Members of the study team reviewed all discharge forms and records of eligible patients, to retrieve information of interest. Data on chronic comorbidities, including the Cumulative Illness Rating Scale (CIRS) [25], frailty measured with the Rockwood Clinical Frailty Scale (CFS) [26], drugs taken before admission, type and dates of anti-SARS-CoV-2 vaccine administration, duration and type of COVID-19-related symptoms before admission, if any, were collected. The CIRS Comorbidity Score (CIRS-CS) was calculated as the sum of ranks of disease severity, from 0 to 4, assigned to each of 14 items representing the main organs and systems involved by chronic diseases [25]. The CIRS Severity Index (CIRS-SI) was calculated as the number of items ranking 3 or 4 in the CIRS scale [25]. CFS ranked from 1 (very fit subject) to 9 (terminal illness) basing on clinical evaluation of each patient’s physical and cognitive performance [26].

Vital signs, lab tests including arterial blood gas analysis and chest CT findings on admission were also considered, if available. The extension of chest CT involvement due to interstitial pneumonia was measured through calculation of the CT visual score, whose procedures are detailed elsewhere [27].

Administration of therapies against COVID-19 (corticosteroids, remdesivir, anti-interleukin-6 drugs), timing to the first RT-PCR test negative for SARS-CoV-2, maximal level of oxygen or ventilatory support needed, duration of hospital stay and need of escalation of care intensity (i.e., transferal to subintensive or intensive care units) were also considered as key elements for defining the clinical course.

The primary endpoint was the presence of severe forms of COVID-19 needing oxygen or ventilatory support and exhibiting a chest CT visual score of at least 5%. Hospital mortality and need of transferal to subintensive or intensive care units were considered as secondary endpoints.

To better describe the clinical characteristics and care needs of patients with COVID-19 breakthrough infection, after careful revision of the clinical presentation, course and resources used during hospital stay, study participants were also classified according to the following scale [22]:

- Asymptomatic for COVID-19 (admission for reasons unrelated to COVID-19, unexpected finding of a positive RT-PCR test);
- Paucisymptomatic for COVID-19 (complex clinical presentation with some symptoms compatible with COVID-19 in presence of another index disease requiring most diagnostic and therapeutical resources);
- Symptomatic for COVID-19 (typical presentation with frequent respiratory failure and positive chest CT findings).

### 2.3. Statistical Analyses

Continuous variables were expressed as median and interquartile range (IQR) and discrete variables as percentage. The clinical and laboratory characteristics of participants were compared after stratification according to chest CT findings (positive versus indeterminate or negative for COVID-19) using Mann–Whitney and chi square tests and, where appropriate, Quade non-parametric Ancova or logistic regression for adjustment for age and sex. A comparison of the clinical and laboratory characteristics was also made according to the categorization as asymptomatic, paucisymptomatic and symptomatic for COVID-19, using Kruskal–Wallis test with significance values adapted to Bonferroni correction for multiple testing, chi-square test, non-parametric Ancova and logistic regression tests for age and sex adjustments. Stepwise logistic regression tests were applied for identifying anamnestic clinical factors independently associated with the presence of interstitial pneumonia on chest CT.

The clinical and laboratory characteristics of participants were also compared after stratification by outcome (hospital mortality) with Mann–Whitney and chi square tests and, where appropriate, Quade non-parametric Ancova or logistic regression for adjustment for age and sex. The same tests were used to compare the characteristics of deceased subjects with and without radiological evidence of pneumonia. Logistic regression models, accounting for all variables with significant differences on descriptive analysis, were then applied to identify factors independently associated with mortality on the whole population of participants and after stratification by age < 85 and ≥85 years old.

The SPSS package (v.28, IBM, Armonk, NY, USA) was used for analyses. *p* values were considered significant when <0.05.

## 3. Results

### 3.1. General Characteristics of Participants and Factors Associated with Positive Chest CT

A total number of 670 patients were admitted to the COVID-19 unit during the study period. Among these, 234 (34.9%) fulfilled inclusion criteria and were classified as having COVID-19 breakthrough infection. Since 5 patients denied informed consent for participation and data treatment, the study population was composed of 229 subjects (124 M, 105 F), with a median age of 81 (IQR 73–88) years old.

Participants had a very high burden of multimorbidity: 210 subjects out of 229 (91.7%) had ≥2 chronic conditions (median 5, IQR 3–7), with a CIRS Comorbidity Score median of 12 (IQR 7–16) and a CIRS Severity Index median of 2 (IQR 1–3). According to the CFS, 58 participants (25.3%) were classified as fit (CFS score 1–3), 30 (13.1%) as pre-frail (CFS score 4), 78 (34.1%) as moderately frail (CFS score 5–6), and 63 (27.5%) as severely frail (CFS score 7–9).

The characteristics of participants, stratified according to positive or negative/indeterminate chest CT findings for COVID-19, are depicted in Table 1. One hundred and thirty-eight patients (60.2%) had chest CT signs compatible with COVID-19 pneumonia. They were younger (age median 79, IQR 71–84, vs. 85, IQR 77–90 years old, *p* < 0.001), less frail (CFS scale median 5, IQR 3–6, vs. 6, IQR 5–7, *p* = 0.005 adjusted for age and sex), but had similar burden of multimorbidity (number of chronic illnesses median 4, IQR 3–6, vs. 5, IQR 3–7, *p* = 0.692 adjusted for age and sex) compared to patients with negative or indeterminate chest CT. The two groups also exhibited substantial differences in lab tests upon admission, but were similar for time elapsed between completion of the vaccine cycle and hospital admission.

Furthermore, 38 patients (16.5%, median age 83, IQR 73–89 years old) were classified as asymptomatic, 69 patients (30.1%, median age 86, IQR 81–90 years old) as paucisymptomatic, and 122 patients (53.4%, median age 78, IQR 70–83) as symptomatic. A comparison of the clinical and laboratory characteristics of patients included in these three categories is shown in Supplementary Material (Table S1).

At a stepwise multivariate logistic regression analysis, accounting for age, sex, CFS, CIRS-CS, chronic illnesses and timing from the last vaccine dose, the only variables with significant association with a positive chest CT were the CFS (OR 0.678, 95% CI 0.573–0.803, *p* < 0.001) and presence of hypertension (OR 1.883, 95% CI 1.049–3.380, *p* = 0.034).

### 3.2. Factors Associated with Mortality

Fifty-eight participants out of 229 (25.3%) died during hospital stay. The clinical characteristics of patients who died, in comparison with survivors, are shown in Table 2.

Patients who died were older, with higher CFS scores and more compromised respiratory exchanges upon admission. However, only 36 of them had positive chest CT for COVID-19. The remaining 22 (38%) deceased without any typical sign of COVID-19 on chest CT. Table 3 shows a comparison of the clinical characteristics between these two groups of subjects.

Logistic regression models, exploring factors associated with mortality in the studied population, are shown in Table 4. Specifically, increasing CFS scores (OR 1.746, 95% CI 1.220–2.500, *p* = 0.002), altered serum levels of procalcitonin on admission (OR 2.569, 95% CI 1.237–5.335, *p* = 0.011) and decreasing values of PaO_2_/FiO_2_ on arterial blood gas analysis on admission (OR 0.989, 95% CI 0.982–0.997, *p* = 0.008) were the only factors independently associated with hospital mortality on a stepwise logistic regression model (Table 4, model 2).

To better explore the role of age on mortality in patients hospitalized with COVID-19 breakthrough infection, the studied population was categorized according to the 85 years old cut-off. Interestingly, the CFS (OR 1.723, 95% CI 1.152–2.576, *p* = 0.008) and admission PaO_2_/FiO_2_ (OR 0.986, 95% CI 0.977–0.996, *p* = 0.005) were independently associated with mortality only in subjects aged <85 years old, but not in subjects aged ≥ 85 years old, where altered serum procalcitonin level was the only parameter independently associated with mortality (OR 3.088, 95% CI 1.389–6.862, *p* = 0.006) (Table 4, models 3 and 4).

## 4. Discussion

Patients admitted with COVID-19 breakthrough infection in an internal medicine ward in Italy in the second half of 2021, during predominance of the SARS-CoV-2 delta variant, were characterized by older age and elevated burden of frailty and multimorbidity. Interestingly, those subjects who were admitted with symptomatic forms of COVID-19 and interstitial pneumonia were on average younger and with lower CFS scores than those with negative or indeterminate chest CT findings. Mortality was not different in these two groups, and was mainly associated with frailty and severity of respiratory impairment in patients < 85 years old, and with serum procalcitonin in patients ≥ 85 years old.

Older frail subjects are more vulnerable to COVID-19 breakthrough infections, because the senescent immune system and chronic activation of inflammatory response typical of frailty syndrome lead to reduced anti-spike antibody titers after vaccination [28,29]. Measures of frailty in nursing home residents, in fact, show an inverse association with antibody titers and duration of the serological response after SARS-CoV-2 vaccination [28,29]. Anti-spike antibody levels are strong predictors of the risk of COVID-19 breakthrough infection and its clinical course in adult subjects with normal immune function [30] and in nursing home residents [31]. Longitudinal studies have also shown a measurable decline of vaccine effectiveness against SARS-CoV-2 infection after six months of completion of the primary vaccination cycle, especially in people aged ≥ 60 years old [32]. All these findings were the bases for the recommendation of receiving a booster dose six months after the completion of the primary vaccination cycle, especially for frail subjects.

In subjects with severe multimorbidity and high CFS scores, COVID-19 may overlap with other cardio-respiratory and neurological diseases, contributing to decompensate them. For example, SARS-CoV-2 infection, even in the absence of clinical and radiological signs of pneumonia, is associated with increased risk of congestive heart failure decompensation [33], chronic obstructive pulmonary disease exacerbation [34], and delirium superimposed on dementia [35,36]. These circumstances generate complex clinical pictures in which it may be particularly difficult to disentangle the contribution of COVID-19 and pre-existing diseases to acute symptoms and radiological findings [22]. Furthermore, the immune response to breakthrough infection after anti-SARS-CoV-2 vaccine, although not optimal, remains measurable and able to modify the natural history of the disease even in the oldest and frailest subjects [6,37,38,39,40]. Indeed, centenarians have shown a better capacity of coping with COVID-19 infection and its consequences, with lower mortality than other groups of geriatric patients [41]. Finally, the apparently reduced frequency of pneumonia in oldest old frail patients hospitalized for COVID-19 may be the result of the increased frequency of RT-PCR testing these patients receive because of precarious clinical conditions and repeated emergency department visits, increasing the probability of detection of asymptomatic and paucisymptomatic infections [42].

In our group of patients with breakthrough infection, mortality was similar in those with and without COVID-19 pneumonia. These findings match those of previous studies reporting a paradoxical increase in mortality for the vaccinated, in comparison with unvaccinated, patients admitted to hospital, due to the average older age and higher burden of frailty and multimorbidity of the vaccinated [43,44,45,46]. In fact, frailty and multimorbidity can influence mortality even when the respiratory involvement in COVID-19 is mild, independently from the intrinsic pathogenicity of SARS-CoV-2 [46,47].

As such, in patients younger than 85 years old with COVID-19 breakthrough infection, mortality was influenced by the severity of respiratory involvement and pre-existing level of frailty (Table 4, Model 3), while, in patients aged 85 or older, by the level of systemic inflammation and the risk of bacterial superinfection, mirrored by serum procalcitonin levels (Table 4, Model 4). In this age range, frailty does not influence mortality in an independent way, probably as a result of a “ceiling” effect due to the diffusion of high CFS scores. Interestingly, these findings are similar to those obtained by our research group in the pre-vaccine era, where serum procalcitonin levels on hospital admission constituted an independent predictor of adverse outcomes only in the oldest old age group [48]. It can be assumed that, in oldest old subjects, the high mortality associated with SARS-CoV-2 breakthrough infection is more influenced by decompensation of pre-existing diseases and concomitant bacterial infections, rather than by COVID-19 itself. SARS-CoV-2 may also involve more severely other organs, and not the lungs, in vaccinated oldest old patients [49].

Conversely, the COVID-19-related factors, including the severity of hypoxemia, presence of ground-glass abnormalities and lung parenchymal infiltrates on chest CT, may still play a relevant prognostic role only in subjects younger than 85. However, the extension of lung parenchymal abnormalities on chest CT, that in previous studies was strongly associated with the severity of respiratory failure and mortality [50,51,52], did not constitute a significant predictor of adverse outcomes in our population with breakthrough infection, suggesting that further research is needed on this issue.

Our study has some limitations. First, we could not perform a reliable comparison among recipients of different vaccines, due to the policy of administration in Italy in 2021, where the mRNA-1273 vaccine was almost exclusively reserved to older subjects with frailty, while ChAdOx1-S and Ad26.COV2.S vaccines were initially reserved to older fit subjects, and then withdrawn from use. Second, we focused only on a period of dominance of SARS-CoV-2 delta variant, preceding the mass administration of vaccine booster doses. Thus, the findings may not be automatically transferred to patients infected by omicron SARS-CoV-2 variant, especially after having received three or more vaccine doses. The single-center design of the study may imply that the findings reflect local policies of hospital admission and management of COVID-19 patients, that are not necessarily the same elsewhere. Finally, the absence of data on unvaccinated patients who were admitted in the same period prevents any comparison between the clinical severity of vaccinated and unvaccinated subjects needing hospital admission.

In spite of this, we provide evidence that patients with COVID-19 breakthrough infection needing hospital admission are mainly geriatric patients with complex clinical needs due to multimorbidity and elevated burden of frailty, and that their mortality remains high even when no signs of pneumonia are present on chest radiology. Thus, in the mass vaccination era, the hospital organization of care should take into account these circumstances to meet the complex needs of these patients [22].

## 5. Conclusions

Patients hospitalized with COVID-19 breakthrough infection in Italy during the dominance of the SARS-CoV-2 delta variant were characterized by older age, multiple comorbidities and elevated burden of frailty. Extreme degrees of frailty, however, were inversely associated with the presence of chest CT signs of interstitial pneumonia. Mortality was associated with frailty and severity of respiratory failure only in subjects younger than 85. Instead, in the oldest old subjects, the only independent prognostic factor was represented by serum procalcitonin levels, suggesting that the high level of mortality in this age range was more associated with decompensation of previous chronic diseases than with the COVID-19 course.

## Figures and Tables

**Table 1:** Comparison of the clinical and laboratory characteristics and outcomes of patients with breakthrough infection categorized according chest CT findings.

	CT Indeterminate or Negative (*n* = 91)	CT Positive (*n* = 138)	*p*	*p* *	*p* * < 0.05 OR
**Demography and personal history**					
Age, years	85 (77–90)	79 (71–84)	**<0.001**	-	
Females, %	53	41	0.090	-	
Chronic illnesses, number	5 (3–7)	4 (3–6)	0.316	0.692	
CFS score	6 (5–7)	5 (3–6)	**<0.001**	**0.005**	
Hypertension, %	56	65	0.164	**0.048**	1.80 (1.00–3.22)
Cardiac disease, %	54	45	0.188	0.369	
Diabetes, %	19	18	0.914	0.968	
Obesity, %	8	14	0.119	0.162	
Dyslipidemia, %	21	19	0.706	0.791	
CKD, %	15	12	0.408	0.672	
Cancer, %	7	5	0.628	0.658	
Dementia, %	32	24	0.186	0.845	
CIRS-CS	13 (8–17)	11 (7–16)	0.164	0.930	
CIRS-SI	2 (2–3)	2 (1–3)	0.095	0.845	
**Vaccination anti-SARS-CoV-2**					
Doses of vaccine received, n	2 (2–2)	2 (2–2)	0.258	0.731	
3 vaccine doses received, %	11	11	0.977	0.807	
Time from second vaccine dose, days	172 (98–219)	181 (104–232)	0.479	0.209	
Time from third vaccine dose, days	22 (14–45)	11 (5–22)	**0.048**	0.103	
BNT162b2 vaccine, %	63	69	0.331	0.278	
mRNA-1273 vaccine, %	27	7	**<0.001**	**0.001**	0.26 (0.11–0.58)
ChAdOx1-S vaccine, %	9	17	0.067	0.129	
Ad26.COV2.S vaccine, %	1	7	**0.049**	**0.044**	12.48 (1.07–146.27)
**Clinical presentation upon admission**					
PaO_2_/FiO_2_, mmHg	329 (293–376)	300 (265–354)	**0.010**	**0.002**	
Duration of symptoms, days	2 (1–4)	5 (3–7)	**<0.001**	**<0.001**	
Fever, %	29	70	**<0.001**	**<0.001**	5.40 (3.00–9.71)
Cough, %	18	43	**<0.001**	**<0.001**	3.49 (1.83–6.64)
Dyspnea, %	31	52	**0.001**	**<0.001**	3.18 (1.74–5.82)
**Blood tests on admission**					
Haemoglobin, g/dL	11.6 (10.7–13.2)	13.2 (11.9–14.3)	**<0.001**	**<0.001**	
Platelet count, 1000/mm^3^	198 (147–264)	184 (155–253)	0.275	0.216	
Neutrophil count, n/mm^3^	4187 (3050–6883)	4804 (3431–7942)	0.085	**0.028**	
Lymphocyte count, n/mm^3^	1003 (771–1556)	928 (620–1403)	0.060	**0.008**	
Creatinine, mg/dL	0.9 (0.7–1.3)	0.9 (0.8–1.3)	0.317	0.113	
C-Reactive Protein, mg/L	39 (15–77)	75 (37–128)	**<0.001**	**<0.001**	
Procalcitonin, ng/mL	0.10 (0.06–0.22)	0.13 (0.07–0.38)	0.098	**0.019**	
D-dimer, ng/mL	1290 (544–2479)	704 (429–1273)	**0.004**	0.098	
CPK, IU/L	86 (45–187)	115 (64–242)	0.056	0.115	
LDH, IU/L	207 (173–236)	273 (220–341)	**<0.001**	**<0.001**	
AST, IU/L	26 (19–35)	32 (24–52)	**<0.001**	**0.001**	
**Clinical course and outcome**					
NIV, %	6	25	**<0.001**	**0.001**	5.41 (2.02–14.52)
IV, %	0	4	0.069	-	
Hospital death, %	24	26	0.746	0.080	
Time before RT-PCR negative, days	13 (7–22)	20 (11–25)	**0.023**	**0.007**	
Hospital stay, days	14 (8–23)	16 (9–28)	0.165	**0.027**	

CT = Computed Tomography; CFS = Clinical Frailty Scale; CKD = Chronic Kidney Disease; CIRS-CS = Cumulative Illness Rating Scale-Comorbidity Score; CIRS-SI = Cumulative Illness Rating Scale-–Severity Index; CPK = Creatine Phosphokinase; LDH = Lactate Dehydrogenase; AST = Aspartate Aminotranspherase; NIV = Non-Invasive Ventilation; IV = Invasive mechanical Ventilation; RT-PCR = Reverse-Transcriptase Polymerase-Chain Reaction. Data are shown as median and IQR or percentages. Crude comparisons were made with Mann–Whitney test or chi-square test, as appropriate. * *p* adjusted for age and sex with Quade non-parametric Ancova or logistic regression. *p* values < 0.05 are indicated in bold.

**Table 2:** Comparison of the clinical and laboratory characteristics of patients with breakthrough infection categorized by hospital outcome (survival vs. death).

	Survivors (*n* = 171)	Dead (*n* = 58)	*p*	*p* *	*p* * < 0.05 OR
**Demography and personal history**					
Age, years	78 (68–86)	86 (82–91)	**<0.001**		
Females, %	46	45	0.856		
Chronic illnesses, number	4 (2–6)	6 (4–7)	**<0.001**	0.205	
CFS score	5 (3–6)	6 (6–7)	**<0.001**	**0.004**	
Hypertension, %	59	69	0.180	0.784	
Cardiac disease, %	46	57	0.137	0.960	
Diabetes, %	19	17	0.802	0.720	
Obesity, %	13	7	0.181	0.738	
Dyslipidemia, %	19	22	0.540	0.504	
CKD, %	11	21	**0.047**	0.337	
Cancer, %	3	14	**0.002**	**0.003**	7.37 (1.95–27.80)
Dementia, %	20	48	**<0.001**	0.069	
CIRS-CS	10 (6–15)	15 (10–18)	**<0.001**	0.070	
CIRS-SI	2 (1–3)	3 (2–4)	**<0.001**	0.062	
**Vaccination anti-SARS-CoV-2**					
Doses of vaccine received, n	2 (2–2)	2 (2–2)	0.195	0.998	
3 vaccine doses received, %	11	12	0.745	0.887	
Time from second vaccine dose, days	174 (100–219)	203 (107–246)	**0.040**	0.300	
Time from third vaccine dose, days	14 (10–35)	16 (10–26)	0.883	0.896	
BNT162b2 vaccine, %	64	72	0.260	0.319	
mRNA-1273 vaccine, %	11	28	**0.003**	0.082	
ChAdOx1-S vaccine, %	19	0	**<0.001**	-	
Ad26.COV2.S vaccine, %	6	0	0.060	-	
**Clinical presentation upon admission**					
PaO_2_/FiO_2_, mmHg	324 (282–375)	276 (227–315)	**<0.001**	**0.001**	
Duration of symptoms, days	4 (1–7)	3 (2–5)	0.381	0.857	
Fever, %	53	55	0.738	0.193	
Cough, %	34	31	0.687	0.537	
Dyspnea, %	38	60	**0.003**	0.064	
**Blood tests on admission**					
Haemoglobin, g/dL	12.6 (11.3–14.0)	12.4 (10.8–13.9)	0.298	0.918	
Platelet count, 1000/mm^3^	194 (150–263)	184 (152–258)	0.390	0.912	
Neutrophil count, n/mm^3^	4438 (3092–6840)	6264 (4009–8588)	**0.004**	**0.024**	
Lymphocyte count, n/mm^3^	1069 (776–1516)	719 (471–1033)	**<0.001**	**0.001**	
Creatinine, mg/dL	0.9 (0.7–1.2)	1.1 (0.7–1.5)	0.055	0.743	
C-Reactive Protein, mg/L	55 (21–90)	90 (38–163)	**<0.001**	**0.004**	
Procalcitonin, ng/mL	0.09 (0.05–0.20)	0.32 (0.11–1.18)	**<0.001**	**<0.001**	
D-dimer, ng/mL	679 (422–1375)	1341 (758–2297)	**<0.001**	**0.045**	
CPK, IU/L	98 (57–200)	125 (46–276)	0.469	0.565	
LDH, IU/L	234 (186–293)	290 (218–352)	**0.002**	**0.001**	
AST, IU/L	29 (22–38)	33 (23–56)	0.064	**0.031**	
**Clinical course and outcome**					
Chest CT positive for COVID-19, %	60	62	0.745	0.080	
NIV, %	14	27	**0.033**	**0.003**	3.66 (1.54–8.69)
IV, %	1	5	0.068	**0.004**	20.96 (2.62–167.37)
Hospital death age < 75, % (N)		2 (1)			
Hospital death age < 85, % (N)		41 (24)			
Hospital death age ≥ 85, % (N)		59 (34)			
Time before RT-PCR negative, days	15 (8–23)	22 (7–31)	0.463	0.724	
Hospital stay, days	15 (8–24)	18 (8–30)	0.459	0.657	

CT = Computed Tomography; CFS = Clinical Frailty Scale; CKD = Chronic Kidney Disease; CIRS-CS = Cumulative Illness Rating Scale-Comorbidity Score; CIRS-SI = Cumulative Illness Rating Scale-Severity Index; CPK = Creatine Phosphokinase; LDH = Lactate Dehydrogenase; AST = Aspartate Aminotranspherase; NIV = Non-Invasive Ventilation; IV = Invasive mechanical Ventilation; RT-PCR = Reverse-Transcriptase Polymerase-Chain Reaction. Data are shown as median and IQR or percentages. Crude comparisons were made with Mann–Whitney test or chi-square test, as appropriate. * *p* adjusted for age and sex with Quade non-parametric Ancova or logistic regression. *p* values < 0.05 are indicated in bold.

**Table 3:** Comparison of the clinical and laboratory characteristics of patients who died with COVID-19 breakthrough infection categorized according to chest CT findings.

	Dead with Negative Chest CT (*n* = 22)	Dead with Positive Chest CT (*n* = 36)	*p*	*p* *
**Demography and personal history**				
Age, years	89 (85–93)	84 (80–90)	**0.019**	-
Females, %	55	39	0.245	-
Chronic illnesses, number	6 (4–7)	6 (4–8)	0.517	0.613
CFS score	7 (6–7)	6 (5–7)	**0.016**	0.214
Hypertension, %	55	78	0.063	0.121
Cardiac disease, %	55	58	0.777	0.937
Diabetes, %	23	14	0.387	0.272
Obesity, %	0	11	0.105	-
Dyslipidemia, %	14	28	0.210	0.353
CKD, %	14	11	0.300	0.308
Cancer, %	14	14	0.978	0.234
Dementia, %	45	50	0.737	0.239
CIRS-CS	13 (10–16)	16 (11–20)	0.251	0.393
CIRS-SI	3 (2–4)	3 (2–4)	0.430	0.424
**Vaccination anti-SARS-CoV-2**				
Doses of vaccine received, n	2 (2–2)	2 (2–2)	0.590	0.975
3 vaccine doses received, %	9	14	0.586	0.838
Time from second vaccine dose, days	177 (112–206)	225 (106–256)	0.059	**0.048**
**Clinical presentation upon admission**				
PaO_2_/FiO_2_, mmHg	300 (260–324)	257 (185–307)	0.060	0.085
Duration of symptoms, days	3 (1–5)	4 (2–6)	**0.024**	0.051
Fever, %	41	64	0.088	0.249
Cough, %	23	36	0.285	0.523
Dyspnea, %	41	72	**0.018**	**0.017**
**Blood tests on admission**				
Haemoglobin, g/dL	11.4 (10.0–12.8)	12.8 (11.4–14.4)	**0.013**	**0.037**
Platelet count, 1000/mm^3^	196 (131–260)	184 (156–253)	0.728	0.820
Neutrophil count, n/mm^3^	6720 (3901–8241)	6194 (4225–9198)	0.917	0.674
Lymphocyte count, n/mm^3^	802 (555–1193)	665 (458–1014)	0.253	0.619
Creatinine, mg/dL	0.9 (0.6–1.5)	1.1 (0.8–1.6)	0.131	0.169
C-Reactive Protein, mg/L	67 (29–125)	109 (41–230)	**0.033**	**0.015**
Procalcitonin, ng/mL	0.33 (0.11–1.66)	0.32 (0.10–1.18)	0.868	0.876
D-dimer, ng/mL	1550 (586–3453)	1297 (764–1982)	0.508	0.851
CPK, IU/L	118 (39–215)	131 (52–367)	0.310	0.473
LDH, IU/L	220 (189–284)	315 (231–374)	**0.005**	**0.006**
AST, IU/L	28 (20–39)	42 (27–77)	**0.024**	**0.040**
**Clinical course and outcome**				
NIV, %	15	33	0.138	0.275
IV, %	0	8	0.164	-
Hospital stay, days	16 (7–32)	20 (8–30)	**0.024**	0.844

CT = Computed Tomography; CFS = Clinical Frailty Scale; CKD = Chronic Kidney Disease; CIRS-CS = Cumulative Illness Rating Scale-Comorbidity Score; CIRS-SI = Cumulative Illness Rating Scale-Severity Index; CPK = Creatine Phosphokinase; LDH = Lactate Dehydrogenase; AST = Aspartate Aminotranspherase; NIV = Non-Invasive Ventilation; IV = Invasive mechanical Ventilation; RT-PCR = Reverse-Transcriptase Polymerase-Chain Reaction. Data are shown as median and IQR or percentages. Crude comparisons were made with Mann–Whitney test or chi-square test, as appropriate. * *p* adjusted for age and sex with Quade non-parametric Ancova or logistic regression. *p* values < 0.05 are indicated in bold.

**Table 4:** Logistic regression models exploring factors independently associated with mortality in the studied population.

	**Odds Ratio**	**95% Confidence Interval**	***p***
***Model 1***			
Age, years	1.080	1.025–1.138	**0.004**
Sex, F vs. M	0.542	0.268–1.096	0.088
Chest CT, positive vs. negative or indeterminate	2.779	1.298–5.953	**0.009**
CFS score	1.740	1.288–2.351	**<0.001**
***Model 2***			
PaO_2_/FiO_2_, mmHg	0.989	0.982–0.997	**0.008**
CFS score	1.746	1.220–2.500	**0.002**
Procalcitonin classes	2.569	1.237–5.335	**0.011**
***Model 3***			
PaO_2_/FiO_2_, mmHg	0.987	0.977–0.996	**0.005**
CFS score	1.723	1.152–2.576	**0.008**
***Model 4***			
Procalcitonin classes	3.088	1.389–6.862	**0.006**

Model 1 accounting for age, sex, chest CT and CFS. Model 2: stepwise method accounting for age, sex, CFS, chest CT findings, cancer, CIRS-CS, CIRS-SI, timing from last vaccine dose, lymphocyte count, neutrophil count, PaO_2_/FiO_2_, LDH, D-dimer, CRP, procalcitonin stratified by classes (class 1 < 0.05 ng/mL, class 2 ≥ 0.05 and <0.5 ng/mL, class 3 ≥ 0.5 and <2 ng/mL, class 4 ≥ 2 ng/mL). Model 3: only patients < 85 years old; stepwise method accounting for age, sex, CFS, chest CT findings, cancer, CIRS-CS, CIRS-SI, timing from last vaccine dose, lymphocyte count, neutrophil count, PaO_2_/FiO_2_, CRP, procalcitonin classes. Model 4: only patients ≥ 85 years old; stepwise method accounting for all variables listed in model 3. CT = Computed Tomography; CFS = Clinical Frailty Scale; CIRS-CS = Cumulative Illness Rating Scale-Comorbidity Score; CIRS-SI = Cumulative Illness Rating Scale-Severity Score; LDH = Lactate Dehydrogenase; CRP = C-Reactive protein. *p* values < 0.05 are indicated in bold.

## Data Availability

Access to data, in anonymous form, can be obtained upon reasonable and motivated request to the corresponding author. The subject entitled for data control and management is the Parma University-Hospital (Azienda Ospedaliero-Universitaria di Parma).

## Supplementary Materials

The following supporting information can be downloaded at: https://www.mdpi.com/article/10.3390/jcm11185442/s1, Table S1: Comparison of the clinical characteristics and outcomes of patients with COVID-19 breakthrough infection, categorized according to the clinical priority of COVID-19 symptoms.
