# Supplementary material for: Multimorbidity and Frailty Are the Key Characteristics of Patients Hospitalized with COVID-19 Breakthrough Infection during Delta Variant Predominance in Italy: A Retrospective Study
Source: J Clin Med. 2022 Sep 16;11(18):5442. doi: 10.3390/jcm11185442 (PMC9503996; doi:10.3390/jcm11185442)
Supplement: Supplementary file 1 [file jcm-11-05442-s001.zip › Supplementary material.pdf]

SUPPLEMENTARY TABLE S1

Comparison of the clinical characteristics and outcomes of patients with COVID-19 breakthrough infection, categorized according to the clinical priority of COVID-19 symptoms.

	**Asymptomatic N.38 (1)**	**Paucisymptomatic N. 69 (2)**	**Symptomatic N.122 (3)**	**p**	**P***	**P<0.05**
**Demography and personal history**						
Age, years	83 (73-89)	86 (81-90)	78 (70-83)	**<0.001**	**-**	
Female sex, %	61	52	38	**0.023**	**-**	
Chronic illnesses, number	4 (2-7)	5 (4-7)	4 (3-6)	**0.020**	0.270	
CFS score	6 (4-7)	6 (5-7)	4 (3-6)	**<0.001**	**0.002**	
Hypertension, %	55	59	65	0.524	0.174	
Cardiac disease, %	53	51	46	0.696	0.705	
Diabetes, %	21	20	16	0.716	0.768	
Obesity, %	11	4	16	0.064	0.143	
Dyslipidemia, %	21	16	21	0.652	0.536	
CKD, %	13	17	11	0.421	0.767	
Cancer, %	3	7	6	0.631	0.655	
Dementia, %	29	36	21	0.084	0.929	
CIRS-CS	10 (6-15)	13 (10-17)	11 (6-16)	**0.042**	0.617	
CIRS-SI	2 (1-3)	3 (2-4)	2 (1-3)	**0.030**	0.698	
**Vaccination anti-SARS-CoV-2**						
Doses of vaccine received, n	2 (2-2)	2 (2-2)	2 (2-2)	0.899	0.758	
3 vaccine doses received, %	11	10	11	0.957	0.784	
Time from last vaccine, days	182 (88-220)	172 (104-217)	182 (104-232)	0.588	0.213	
**Clinical presentation upon admission**						
PaO_2/FiO_2, mmHg	374 (333-408)	319 (276-374)	298 (262-343)	**<0.001**	**<0.001**	(1) vs (2) vs (3); (2) vs (3)
Duration of symptoms, days	1 (0-5)	2 (1-4)	5 (3-7)	**<0.001**	**<0.001**	(3) vs (1) vs (2)
Fever, %	8	38	76	**<0.001**	**<0.001**	(1) vs (2) vs (3); (2) vs (3)
Cough, %	0	28	47	**<0.001**	**<0.001**	(1) vs (2) vs (3); (2) vs (3)
Dyspnea,%	3	48	54	**0.001**	**<0.001**	(1) vs (2) vs (3); (2) vs (3)
Chest CT positive for COVID-19, %	0	25	99	**<0.001**	**<0.001**	(1) vs (2) vs (3); (2) vs (3)
**Blood tests on admission**						
Haemoglobin, g/dl	11.5 (10.5-13.1)	11.6 (10.7-13.0)	13.6 (12.1-14.4)	**<0.001**	**<0.001**	(3) vs (1) vs (2)
Platelet count, $1000/mm^3$	218 (189-265)	189 (131-270)	184 (155-243)	**0.049**	0.092	
Neutrophil count, n/mm^3	5039 (2878-6785)	4437 (3244-7582)	4740 (3358-7874)	0.508	0.308	

Lymphocyte count, n/mm^3	1130 (794-1767)	1012 (769-1520)	866 (606-1333)	**0.013**	**0.001**	(3) vs (1) vs (2)
Creatinine, mg/dl	0.7 (0.6-1.1)	0.9 (0.7-1.4)	1.0 (0.8-1.3)	0.050	0.059	
C-reactive protein, mg/L	28 (13-52)	51 (21-91)	75 (39-131)	**<0.001**	**<0.001**	(1) vs (2) vs (3); (2) vs (3)
Procalcitonin, ng/ml	0.08 (0.05-0.12)	0.11 (0.07-0.36)	0.13 (0.07-0.40)	**0.007**	**0.004**	(1) vs (3)
D-dimer, ng/ml	1345 (603-3596)	1105 (421-2004)	692 (432-1240)	**0.007**	0.062	
CPK, IU/L	80 (45-207)	100 (44-219)	115 (69-252)	0.084	0.238	
LDH, IU/L	215 (174-266)	211 (175-251)	275 (220-346)	**<0.001**	**<0.001**	(3) vs (1) vs (2)
AST, IU/L	26 (18-35)	25 (20-36)	33 (26-55)	**<0.001**	**0.001**	(3) vs (1) vs (2)
**Clinical course and outcome**						
NIV, %	0	8	28	**<0.001**	**<0.001**	(3) vs (1) vs (2)
IV, %	0	0	4	0.113	0.197	
Hospital death, %	16	29	26	0.316	0.087	
Time before RT-PCR negative, days	7 (7-19)	16 (8-23)	20 (11-28)	**0.015**	**0.007**	(1) vs (3)
Hospital stay, days	12 (7-22)	16 (10-24)	16 (8-28)	0.279	0.122	

CFS=Clinical Frailty Scale; CKD=Chronic Kidney Disease; CIRS-CS=Cumulative Illness Rating Scale-Comorbidity Score; CIRS-SI=Cumulative Illness Rating Scale-Severity Index; CT=Computed Tomography; CPK=Creatine Phosphokinase; LDH=Lactate Dehydrogenase; AST=Aspartate Aminotranspherase; NIV=Non-Invasive Ventilation; IV=Invasive mechanical Ventilation; RT-PCR=Reverse-Transcriptase Polymerase-Chain Reaction. Data are expressed as median and interquartile range or percentage, as appropriate. P calculated with Kruskal-Wallis test, with significance values adapted basing on Bonferroni correction for multiple testing or logistic regression. *P adjusted for age and sex with Quade non-parametric Ancova or logistic regression, where appropriated. P values <0.05 are indicated in bold.
